# Impact of Gut Microbiome Manipulation in 5xFAD Mice on Alzheimer’s Disease-Like Pathology

**DOI:** 10.3390/microorganisms9040815

**Published:** 2021-04-13

**Authors:** Malena dos Santos Guilherme, Vu Thu Thuy Nguyen, Christoph Reinhardt, Kristina Endres

**Affiliations:** 1 Department of Psychiatry and Psychotherapy, University Medical Center of the Johannes Gutenberg-University Mainz, 55131 Mainz, Germany; malena-guilherme@gmx.de (M.d.S.G.); VuThuThuy.Nguyen@unimedizin-mainz.de (V.T.T.N.); 2 Center for Thrombosis and Hemostasis (CTH), University Medical Center of the Johannes Gutenberg-University Mainz, 55131 Mainz, Germany; Christoph.Reinhardt@unimedizin-mainz.de; 3 German Center for Cardiovascular Research (DZHK), University Medical Center of the Johannes Gutenberg-University Mainz, 55131 Mainz, Germany

**Keywords:** microbiota, antibiotics, probiotics, intestines, neurodegenerative diseases, diabetes mellitus, receptor for advanced glycation end products, glucagon

## Abstract

The gut brain axis seems to modulate various psychiatric and neurological disorders such as Alzheimer’s disease (AD). Growing evidence has led to the assumption that the gut microbiome might contribute to or even present the nucleus of origin for these diseases. In this regard, modifiers of the microbial composition might provide attractive new therapeutics. Aim of our study was to elucidate the effect of a rigorously changed gut microbiome on pathological hallmarks of AD. 5xFAD model mice were treated by antibiotics or probiotics (*L. acidophilus* and *L. rhamnosus*) for 14 weeks. Pathogenesis was measured by nest building capability and plaque deposition. The gut microbiome was affected as expected: antibiotics significantly reduced viable commensals, while probiotics transiently increased *Lactobacillaceae*. Nesting score, however, was only improved in antibiotics-treated mice. These animals additionally displayed reduced plaque load in the hippocampus. While various physiological parameters were not affected, blood sugar was reduced and serum glucagon level significantly elevated in the antibiotics-treated animals together with a reduction in the receptor for advanced glycation end products RAGE—the inward transporter of Aβ peptides of the brain. Assumedly, the beneficial effect of the antibiotics was based on their anti-diabetic potential.

## 1. Introduction

The eliciting factors for sporadic Alzheimer’s disease (AD) are still to be identified. Several genetic and environmental risk factors have been identified such as the ApoE4 allele or diabetes (for example [1,2]) that contribute in different proportion to the overall risk load of the individual. Within the last decade, a new approach was introduced by considering the human microbiome as one of the disease modifiers if not even a trigger [3]. Differences in the gut microbiome as well as other microbiomes such as the salivary one have been studied in AD patients and aging cohorts [4,5,6,7]. It is tempting to assume that, for example, the gut–brain axis might translate such changes within distinct microbial communities towards the brain of the host and thereby initiate, ameliorate or aggravate pathological mechanisms within the central nervous system [8]. For example, neurotrophic factors such as BDNF or dendritic spine formation within the hippocampus have been shown to be associated with presence of a gut microbiome or its composition (for a recent review see [9]).

Interestingly, the relation between the microbiome and this devastating disease might be double-edged. One of the major hallmarks of AD are the neurotoxic Aβ peptides derived by proteolytic processing of the amyloid precursor protein (AβPP). These peptides have been suggested to act in an anti-microbial manner, and in vitro as well as in vivo effects on selected bacteria as well as viral species and microbial communities have been reported [10,11,12,13]. Recently, the occurrence of Aβ has been demonstrated in the saliva of AD model mice and comparably in samples derived from human patients [14]. Additionally, App NL-G-F but not APP/PS1 mice displayed an altered oral microbiome. Another study revealed that in C57BL/6J wild type mice a single oral administration of Aβ altered the gut microbiome after inoculation for the time of a single gut passage only [15].

This might hamper the analysis of the microbiome and might explain why a conclusive picture about the typical “AD-microbiome” still is lacking. Some bacterial commensals seem to at least be repeatedly identified such as *Alistipes*, *Blautia*, *Odoribacter*, *Ruminococcus*, and *S24-7* [16].

An approach to unravel the effect of the microbiome on pathogenesis is the manipulation of the microbiome and here, most work has been focused on the gut commensal community. Under germ-free conditions, for example, APP/PS1 mice showed an attenuated pathology [17]. A six-month treatment regime with an antibiotics cocktail did not reduce microbial abundance in general (measured by 16SrRNA copy number, [18]) but affected the community as α-diversity was lowered. This was accompanied by a more than two-fold decrease in combined cortical and hippocampal Aβ plaque burden in male antibiotics-treated mice as compared to vehicle-receiving animals. As a potential explanation for this beneficial effect, the authors demonstrated elevations of circulating cytokines such as CCL11, and more importantly increased ramification of microglia surrounding the senile plaques within the brain.

Furthermore, pre- and probiotics have been tested with regard to an anti-AD potential. In a rat model, where AD-like symptoms were evoked by treatment with D-galactose and Aβ, oligosaccharide extracted from *Morinda officinalis* (OMO) was administered orally for four weeks [19]. This resulted in attenuation of learning and memory deficits of the AD model as shown by Morris Water Maze test, recovering of control cytokine levels in blood and decreased levels of Aβ1-42 and Tau proteins. By administration in an inflammatory bowel disease model, OMO proved its prebiotic character. Probiotic *Lactobacillus* strains rescued the rough eye phenotype that is found in AD-induced *Drosophila* [20]. ProBiotic-4, a probiotics mixture of *Bifidobacterium lactis*, *Lactobacillus casei*, *Bifidobacterium bifidum*, and *Lactobacillus acidophilus* was capable of attenuating disruption of the intestinal barrier and blood–brain barrier and improved memory deficits in aged SAMP8 mice [21]. This inbred strain shows an accelerated aging phenotype and resembles several pathological hallmarks of AD (for example [22]).

Aim of our study was to compare the probable efficacy of either antibiotics treatment or probiotics administration on AD-like pathology within the 5xFAD mouse model in a direct, parallel comparison approach. The 5xFAD mouse model is a rather aggressive and fast developing model with the occurrence of first plaques at the age of 1.5 months [23]. Therefore, we chose to start the treatment paradigm at an age of four weeks and to proceed up to the age of 18 weeks where first behavioral deficits can be observed.

## 2. Materials and Methods

### 2.1. Animals

B6SJL-Tg(APPSwFlLon,PSEN1*M146L*L286V)6799Vas/Mmjax (5xFAD) mice (Jackson Lab, Bar Harbor, Maine, USA) were stably crossbred on a C57BL/6J background. The animals were group-housed (three to five animals) until the start of the experiment, then they were single-caged to prevent coprophagy. Animals were kept with a 12 h day/night cycle; food and water were available *ad libitum*. All procedures were performed in accordance with the European Communities Council Directive regarding care and use of animals for experimental procedures and were approved by local authorities (Landesuntersuchungsamt Rheinland-Pfalz; approval number G17-1-035, approval date June 2017). Germ-free C57BL/6J that were used for assessing viability of probiotic bacteria after stomach passage were maintained as colonies in sterile flexible film mouse isolator systems at the Translational Animal Research Center (TARC) [24,25]. The germ-free status of the mice was verified every second week by 16S rDNA PCR and by bacterial culture testing.

### 2.2. Treatment of Mice via Drinking Water

All animals received food (Ssniff Spezialdiäten GmbH, Soest, Germany) and tap water *ad libitum*. At an age of 4 weeks, animals received an antibiotics mixture as described previously [18]. We assumed a maximal gavage volume of 500 µL and adopted for a mean drinking volume of 4 mL the following concentrations: gentamicin (0.1251 mg/mL), vancomycin (0.0635 mg/mL), metronidazole (0.25 mg/mL), neomycin (0.0635 mg/mL), ampicillin (0.1251 mg/mL), kanamycin (0.3753 mg/mL), colistin (7,506,000 U/mL), and cefoperazone (0.1251 mg/mL). Metronidazol, vancomycin, ampicillin, cefoperazone, and kanamycin were obtained from Cayman (Ann Arbor, Michigan, MI, USA), gentamicin and neomycin from Sigma; colistin from J&K Scientific (Beijing, China). The animals were kept for two weeks on the high dosage, then dosage was reduced to 1/50 and animals maintained on the treatment for a further 12 weeks. Control animals received tap water only, probiotics-treated animals received 10^9^ CFU/mL (OptiBac for those on antibiotics, OptiBac Probiotics HQ Wren Laboratories Ltd., Hampshire, UK, contains *L. acidophilus* and *L. rhamnosus*). The water was exchanged two-times a week, animals were treated for 14 weeks in total. Consumption of drinking water was indistinguishable between the three groups (controls: 4.6 ± 1.2 mL, antibiotics group: 4.8 ± 1.2 mL, probiotics group: 4.2 ± 0.7 mL).

### 2.3. Viability of Lactobacillaceae in Stomach Content

From germ-free mice, 10 mg of stomach content were collected during sacrifice, dissolved in 750 µL PBS, and incubated for 10 min at 600 rpm at room temperature. OptiBac powder, which served as the probiotic treatment, was dissolved in PBS to reach 400 CFU/mL. Equal volumes of bacterial suspension were diluted 1:1 either with PBS (positive control) or with stomach content. Stomach content diluted 1:1 with PBS served as a negative control. All samples were incubated for 70 min (estimated stomach passage of mice, [26]) at 500 rpm and 37 °C.

### 2.4. Bacteria Plating and CFU Assessment

Samples were diluted appropriately with sodium chloride and plated on 3M^TM^ Petrifilm plates (3M Deutschland GmbH, Neuss, Germany) for *Lactobacillaceae* and *Enterobacteriaceae*. Colony forming units were counted after 20 h incubation at 37 °C.

### 2.5. Nest Building Test

The nest building test was performed as described previously [27]. In brief, animals were habituated to a special nesting material and received 10 g of nesting material for overnight nest building. The next morning before 9:00 a.m., nest quality was scored and material not integrated into the nest was weighed.

### 2.6. Sacrifice and Tissue Preparation

Animals were weighed, anesthetized with isoflurane (Piramal, Mumbai, India), and sacrificed by decapitation. Truncal blood was collected and blood sugar immediately measured from one droplet by a blood glucose guide meter (Accu-Chek, Roche, Basel, Switzerland). The residual blood was used for serum preparation by two consecutive centrifugation steps (first time at 680× *g*, second time at 15,680× *g*, both for 10 min at 10 °C). Thymus, adrenal glands, liver, spleen, epididymal fat and colon were dissected and weighed or measured (colon length). The brain tissue was collected as hemispheres without olfactory bulbs and the left hemisphere drop-fixed in 4% formaldehyde for 24 h.

### 2.7. Immunohistochemistry and Densitometric Analysis

IHC sections were stained with anti-APP antibody 6E10 (Covance) as described previously [28]. For the densitometric analysis, two sections per mouse were used (total magnification of 40×). Five areas were determined to be measured. All areas were corrected for the value of the background area. For cortical tissue, two distinct areas were analyzed and mean value of both measures was used. Experimenters were blinded for the treatment of the mice during the analysis. Microscopic pictures of the IHCs were acquired by an EVOS XL microscope (Life Technologies, Darmstadt, Germany). AIDA image analyzer 4.26 software (Raytest, Straubenhardt, Germany) was used for quantitative analysis.

### 2.8. Measurement of Serum Insulin and Glucagon

Serum was prepared from truncal blood by two consecutive centrifugation steps after a minimum clotting time of 45 min. Samples were stored at −80 °C until further usage. 10 (Insulin ELISA) and 25 (Glucagon ELISA) µL were subjected to analysis following the vendor’s recommendations (Mercodia, Uppsala, Sweden).

### 2.9. Western Blotting

Right brain hemispheres were homogenized in Tris HCl buffer supplemented with proteinase inhibitor cocktail (Roche complete mini) by using the Tissue Lyzer (Qiagen, Hilden, Germany). Proteins (40 µg per lane, determined by Roti Nanoquant reagent) were separated on 10% SDS PAA gel and blotted onto nitrocellulose. Non-specific binding was blocked with 0.2% I-Block (Thermo Fisher Scientific, Waltham, MA, USA) solution including 0.05% Tween20. Primary antibodies were as follows: anti-RAGE (1:1000, Santa Cruz Biotechnology, Dallas, TX, USA) and XBP-1 (1:1000, Abcam, Cambridge, UK). As a loading control, GAPDH was detected (14C10, Cell Signaling, Danvers, MA, USA). Blots were incubated with respective secondary antibody coupled with horseradish peroxidase (Thermo Scientific, Karlsruhe, Germany) and signals obtained by administration of SuperSignal West Femto chemiluminescent substrate (Thermo Scientific, Karlsruhe, Germany). Chemiluminescence signals were captured using a CCD-camera imaging system (Raytest, Straubenhardt, Germany) and densitometric analysis performed by using AIDA image analyzer 4.26 software (Raytest, Straubenhardt, Germany).

### 2.10. Statistical Analysis

For comparisons one-way analysis of variance (ANOVA) was performed followed by a post hoc test as indicated. *p*-values < 0.05 were considered statistically significant and results were presented as mean + SEM. Data analyses were performed using GraphPad Prism 8 (Graph Pad Software, La Jolla, CA, USA).

## 3. Results

### 3.1. Manipulation of the Gut Microbiome of 5xFAD Mice

**Figure 1:**
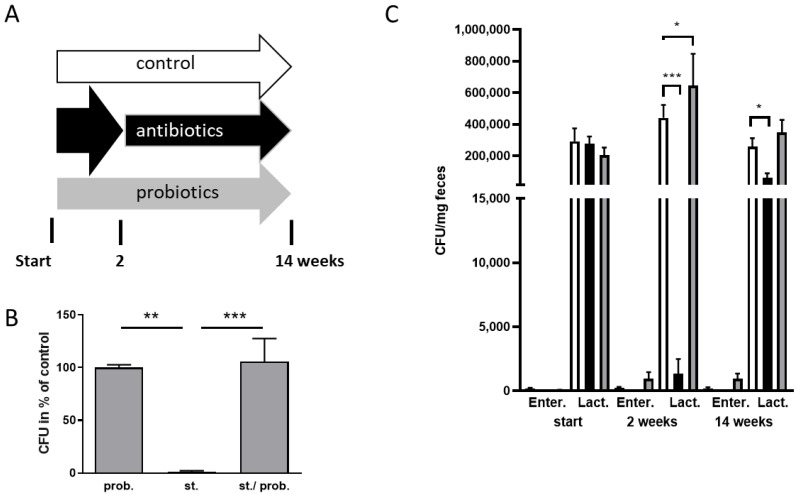
Manipulation of gut microbiota by application of antibiotics and probiotics with drinking water in 5xFAD mice. Male mice aged 4 weeks were subjected to three treatment groups: control, antibiotics or probiotics (*n* = 8 for control, *n* = 7 for pro- and antibiotics each). Within the antibiotics group two weeks of high dosage and 12 weeks of low maintenance dosage were applied via the drinking water (**A**, scheme). (**B**) To control for general viability of the orally administered probiotics within the intestinal passage, stomach contents from germ-free, gnotobiotic mice (*n* = 18) were spiked with a defined amount of the probiotic bacteria, incubated for 70 min and plated on *Lactobacillaceae*-specific plates. As a positive control, probiotic bacteria in solvent were used, (prob., *n* = 8); stomach contents from the animals diluted with solvent served as a negative control (st.). Colonies observed after 20 h were counted (colony forming units, CFU) and normalized to the positive control. (**C**) Feces from the animals of the three treatment groups were collected at the indicated time points, diluted with sodium chloride and plated on *Enterobacteriaceae*- and *Lactobacillaceae*-specific plates. CFU were related to the used fecal material amount. Data are presented as mean + SEM. Statistical analysis was performed by one-way ANOVA with the appropriate post-test (B: Tukey’s, C: Fisher’s LSD; *, *p* < 0.05; **, *p* < 0.01; ***, *p* < 0.001).

The reduction of the gut microbiome within the APP/PS1 AD mouse model has been shown to exert a beneficial effect on hallmarks of the disease-like state within the animals [18]. All transgenic mouse models of AD display unique characteristics e.g., concerning the timeline of symptoms or severity of symptoms. We therefore wanted to analyze if the comparably aggressive 5xFAD mouse model might also benefit from such treatment. We modified the treatment paradigm by administering two weeks a high dosage and then providing a low maintenance dosage for another 12 weeks. Start point of the treatment were male mice aged four weeks (schematic of the experiment provided in Figure 1A). The relatively early age was chosen as first plaque depositions can already be observed at about 1.5 months of age in this mouse line [23]. To assess the influence of bacteria assumed to exert a probiotic function, 5xFAD mice were also treated with a mixture of *L. acidophilus* and *L. rhamnosus* (animals designated as probiotics group). As all treatments were conducted via drinking water and with this by oral passage, firstly the survival rate of *Lactobacilli* was tested using the stomach contents of germ-free mice (Figure 1B). Bacteria were added to stomach contents or PBS and plated after 70 min incubation at 37 °C, which simulated time to enter the intestine in mice [26]. CFU obtained for bacteria incubated with stomach content was indistinguishable from numbers of control (PBS)-treated bacteria (*p* = 0.97). Therefore, these probiotic bacteria were sufficiently viable to enter and colonize the intestine after ingestion.

To monitor effect of the treatment by anti- and probiotics, fecal samples were analyzed after two weeks and at the end of the experiment (Figure 1C). As representatives, cultivatable *Enterobacteriaceae* and *Lactobacillaceae* were quantified by using specific plates. The latter family also served as the proof of probiotics colonization. Before start of the treatment, colonies from all three treatment groups were comparable in regard to both bacterial families. After two weeks, the high dosage of antibiotics resulted in a reduction of both, reaching significance for the *Lactobacillaceae*. In the probiotics-treated groups, *Lactobacillaceae* were significantly elevated, demonstrating a successful colonization by exogenously added members of this family. At the end of the treatment period, the antibiotics effect was still present even if the low dosage allowed growth of more bacteria than after the high dosage application. Within the probiotics-treated mouse group, an increase in *Lactobacillaceae* was still measured; however, this did not reach significance (control: 259,286 CFU; probiotics group: 350,333 CFU; *p* = 0.298).

### 3.2. Effect of Gut Microbiome Alteration on Pathological Hallmarks of 5xFAD Mice

**Figure 2:**
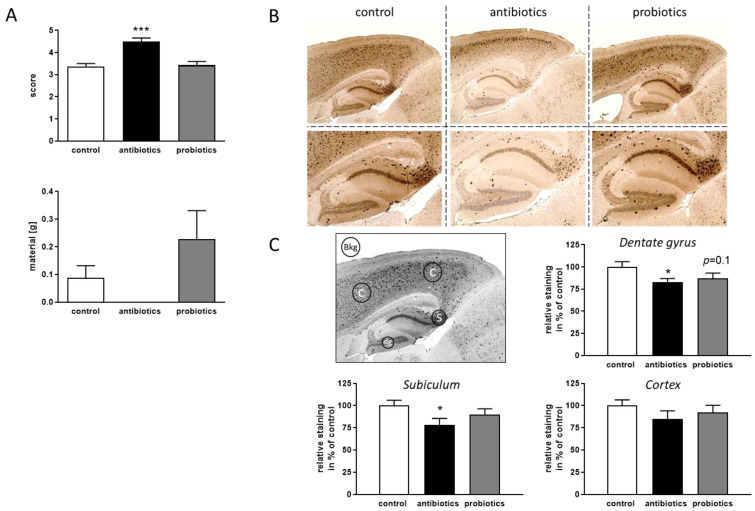
Influence of gut microbiota manipulation on pathological hallmarks of 5xFAD mice. (**A**) Within the 14th week of treatment, nest-building ability as a proxy for hippocampal function was assessed: quality of the nests (score) as well as the amount of not integrated material were measured. Statistics were performed with a one-way ANOVA and Tukey’s post-test (***, *p* < 0.001). (**B**) After 14 weeks of treatment, animals were sacrificed and sagittal sections of the brain were stained for Aβ-depositions. The area of the hippocampus is shown magnified within the exemplary pictures. (**C**) Aβ-depositions were quantified densitometrically in the indicated brain regions of two independent slices per animal and corrected for background staining intensity (Bkg.). Pixel measures were normalized to the mean of control-treated animals and are depicted as percentage. Data are presented as mean + SEM. (Multiple t-test; *, *p* < 0.05; one sample from the antibiotics-treated animals was not analyzed due to technical reasons).

Assessing the quality of the nest built by the mouse, reports on well-being of the animal and on integrity of the hippocampus as has been demonstrated i.e., by hippocampal lesion experiments [29]. When scoring the quality of the nests shortly before the end of the experiment (14th week), better nests were obtained for antibiotics-treated 5xFAD mice and the integration of material was also optimized—even if the latter did not reach statistical significance (Figure 2A). An analysis of Aβ deposition in the hippocampal area confirmed the finding of ameliorated pathology (Figure 2B,C), while in cortical areas only subtle, non-significant decrease of deposited material was observed, the dentate gyrus and subiculum both showed reduced staining intensities. Treatment with probiotics elicited no change in any of the investigated regions.

### 3.3. Antibiotics-Treatment of 5xFAD Mice Reveals Anti-Diabetic Properties

**Figure 3:**
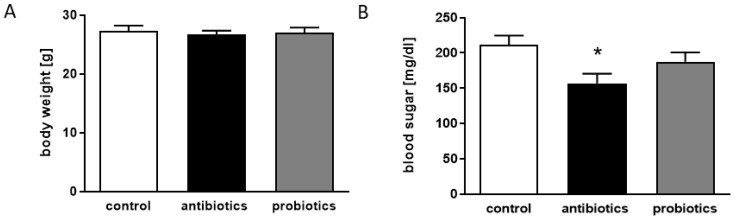
Antibiotics reduce blood sugar level of 5xFAD mice. (**A**) Body weight of the mice was measured after 14 weeks of the respective treatment. (**B**) Samples from truncal blood collected during sacrifice were used for quantification of blood sugar levels. Data are given as mean + SEM. Statistical analysis was performed by one-way ANOVA with Tukey’s post-test (*, *p* < 0.05).

Both, depletion of the microbiome as well as the administration of probiotics, might reveal non-brain effects—e.g., on tissues relevant to immunity or hormone signaling. Therefore, additional parameters were assessed with sacrifice, such as thymus and spleen weight, adrenal gland weight, weight of abdominal fat pads (Supplementary Materials Figure S1) or body weight (Figure 3A). None of these were affected, either by antibiotics nor by probiotics when measures were compared to control animals. However, blood sugar levels were specifically decreased in the antibiotic-receiving mice (Figure 3B). As sacrifice of the mice was performed around 10 a.m., it must be assumed that the mice were at a fasting state (lights on in the animal facility was at 6 a.m.).

**Figure 4:**
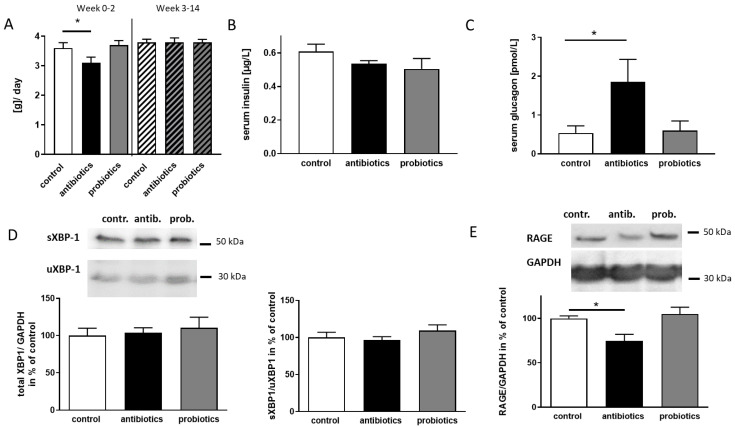
Effect of antibiotics on blood glucose homeostasis and insulin-dependent pathways. (**A**) Food consumption of the mice was measured twice a week. Columns without pattern show the food intake within the first two weeks, patterned columns show the food consumption within the weeks 3 to 14. (**B**) Serum insulin was measured by ELISA using 10 µL sample in duplicate. For glucagon (**C**) 25 µL were used in technical duplicates. (**D**) Splice variants of XBP-1 (S: spliced; u: unspliced) were quantified by Western blotting and ECL-based signal development due to appropriate HRP-labelled secondary antibody. (**E**) RAGE and GAPDH, which served as a loading control, were visualized as described (*n* ≥ 5 per group for Western blots and ELISAs). Mean + SEM are presented. Statistics were performed with one-way ANOVA and Dunnett’s post-test (*, *p* < 0.05).

To ascertain that the observation was not related to altered feeding behavior, food consumption within the first two weeks and within the last 12 weeks of treatment was compared between control animals and treated groups (Figure 4A). Probiotics had no effect on food consumption overall. Antibiotics reduced food intake during the period of high dosage treatment by 0.5 g per day. However, within the subsequent 12 weeks on low dosage, this reduced level returned to control levels. Therefore, general hypophagia can be excluded as a cause for the observed reduction in blood glucose level.

For lean mice, a drop in blood glucose together with rise in glucagon and decrease in insulin levels has to be expected [30], while in obese or insulin resistant mice, for example, the fasting-induced decrease in serum glucose is blunted. When measuring insulin in both, samples from antibiotics- and probiotics-treated mice, a decrease in insulin was assessed that did not reach statistical significance (*p* = 0.426 and 0.209, Figure 4B). For glucagon, a significantly higher mean value was obtained in antibiotics-receiving animals as compared to control 5xFAD mice, while probiotics showed no effect. We therefore interpreted the observed effect as an anti-diabetic shift in blood glucose control as due to the 14 weeks administration of antibiotics.

Several pathways involved in AD pathology have been considered to depend on insulin [31], and type II diabetes is one of the risk factors for this disease in humans [32]. An altered microbiome might have favorable effects on each of the conditions, but also on the cross-talk between AD and a diabetic phenotype (reviewed in [33]). To understand if any of the known molecular mechanisms might be involved in the here observed pathology-ameliorating effects, we exemplary investigated XBP-1 and RAGE. The IRE1 α/XBP-1 pathway is activated by ER stress as, for example, evoked by unfolded proteins and results in a spliced mRNA. The resulting protein (sXBP-1) is double the size of the one encoded by the unspliced mRNA (uXBP-1, 30 kDa, [34]). Components of this pathway have been shown to function at an increased basal level in the presence of insulin resistance and hyperinsulinemia [35]. Moreover, sXBP-1 has been shown to act as a transcriptional activator of the α-secretase ADAM10 [36]. Quantitation of both, s- and u-XBP-1 revealed no elevated protein amounts—neither by anti- nor by probiotics treatment (Figure 4D). Moreover, no alteration in the ratio between both could be observed.

The formation and accumulation of advanced glycation end products (AGEs) is part of the normal aging process but occurs accelerated under diabetic conditions [37]. AGEs bind to RAGE, the receptor for AGEs and thereby lead to sustained NFκB activation and inflammation (for a review see [38]). Moreover, RAGE is also responsible for brain-directed transport of Aβ peptides from the periphery as a counterpart to LRP1 [39]. In antibiotics-treated 5xFAD mice, a reduction of RAGE by an amount of about 30% was assessed, while probiotics did not affect the expression of the receptor (Figure 4E).

## 4. Discussion

Within the 5xFAD mouse model, treatment with antibiotics over a period of 14 weeks, starting at an early pathological stage, resulted in an ameliorated pathogenesis while probiotics administration remained without effect. In addition, the antibiotics led to an anti-diabetic shift that also was accompanied by decreased expression of RAGE, which might have contributed to the beneficial property of the intervention.

For the probiotics treatment, we obtained a statistically significant increase in *Lactobacillaceae* in the early phase of the treatment. The selective plates used in the experiments allowed growth of *Lactobacillaceae*, the family that also includes the administered bacterial species *L. acidophilus Rosell-52* and *L. rhamnosus Rosell-11*. It can therefore be assumed that colonialization with the exogenously applied probiotics was successful, even if their amount was not specifically addressed. After 14 weeks of continuous administration, no significantly elevated *Lactobacillaceae* amount was noted anymore. However, it has to be stated that administration of probiotics not only affects the amount of the added species but also has far-reaching consequences for the whole community. This probiotics-driven effect on the gut microbiome has been demonstrated for several species and bacterial strains before [40,41,42]. Especially, beneficial effects against enteropathogens can be assumed by the potential to supersede these from the surface of host cells (demonstrated on Caco-2 cells, [43]). Moreover, additional positive effects of probiotic strains directly exerted on the hosts’ intestinal cells have been reported, such as electrolyte absorption, prevention of intestinal damage by TNBS, and downregulation of Glut2 using rodent or cellular models [44,45,46]. Therefore, we in principle hypothesized a disease-ameliorating effect in 5xFAD mice by the administered probiotics, as has been shown for other models before. In senescence accelerated mouse models and in several attempts using Aβ-injection or other pharmacologically-derived rat models of AD, such probiotics improved memory deficits and ameliorated neuroinflammation [21,47,48,49,50,51,52]. In the murine model strain APP/PS1, recent publications also reported that *L. plantarum* administration (alone or in combination with *B. bifidum*, [53,54]) alleviated pathological symptoms. In one of the studies, however, the effect of *L. plantarum* was mainly noted to be an augmentation when administered together with memantine by inhibiting synthesis of the gut microbial metabolite trimethylamine-N-oxide, and decreasing neuroinflammation [54]. Within another study, the beneficial effect regarding cognitive performance was mainly seen in the approach where two strains (*L. plantarum* and *B. bifidum*) were combined [53].

SLAB51, a formulation made of nine live bacterial strains (containing *L. acidophilus*), was also able to positively affect several disease-associated pathways in 3xTg AD model mice [55]: for example, SIRT1 activity was increased in brain homogenates, as was the activity of antioxidant enzymes such as GST. However, cognitive improvement has not been shown in the respective investigation. In a meta-analysis, it was shown for 12 out of 16 studies, in which a direct comparison between single strain administration and treatment with probiotic mixtures was reported, that mixtures were more efficient in providing health-promoting effects [56]. In our study, we treated the 5xFAD mice with a mixture of two strains, *L. acidophilus Rosell-52* and *L. rhamnosus Rosell-11*, and at least *L. acidophilus* has been used successfully as a component of such mixtures before. Nevertheless, no significant effect on behavior, plaque deposition or metabolism was obtained in 5xFAD mice. Potentially, the concerted interplay of another probiotic here was missing that could not be compensated sufficiently by *L. rhamnosus*. Usage of probiotics in AD patients still is only a future perspective and data from human studies up to now have not been convincing: a small study in 30 patients per group described a positive effect of a *L. acidophilus*-containing probiotic mix on cognitive function and some metabolic parameters [57]. A recent meta-analysis, however, concluded from three randomized clinical trials, suiting the filter criteria and involving 161 individuals with AD, no benefit for cognitive function (all studies used *Lactobacillus* and *Bifidobacterium* strains, *L. acidophilus* contained in all, [58]). Interestingly, treatment resulted in improved plasma triglycerides and insulin resistance.

For antibiotics-based depletion of the microbiome of 5xFAD mice, a treatment regimen was applied that has been reported before [18] with slight deviations, namely a shorter treatment period (14 weeks instead of 6 months) and delivery via drinking water instead of gavage from the very beginning. After two weeks of high dose antibiotics, a strong reduction in viable *Lactobacilli* occurred, which flattened out with the end of the experiment. This coincides with the data obtained for APP/PS1 mice at the end of the treatment reported by Minter and colleagues [18]. No general reduction of prokaryotic fecal abundance as measured by PCR copy number was observed, but rather a shift in the composition. Moreover, a reduction in relative abundance in OTU reads for *Lactobacillaceae* was also found but it was not reported if this difference was statistically significant. A limitation of our study might be seen within the lack of a detailed microbiome analysis via sequencing which does not allow a direct comparison with the study initially describing, for example, the antibiotics treatment. However, our aim was more to investigate the outcomes concerning pathology in the model mice.

There are controversial opinions on the usage of antibiotics cocktails in comparison to germ-free raised animals. Both methods have their pros and cons as e.g., germ-free conditions might impact gut development (for example [25,59,60]) but antibiotics might not deplete bacterial commensals completely and have side effects [61]. By comparing both attempts in 5xFAD mice, both strategies revealed lowered plaque burden in the hippocampus in mice and ameliorated behavioral deficits, however, microglial activation was selectively found in germ-free raised animals [62]. The antibiotics treatment regimen differed from the here used one (containing 1 mg/mL vancomycin, 1 mg/mL cefoxitin, 1 mg/mL gentamicin and 1 mg/mL metronidazol for two months). As an investigation in APP/PS1 mice demonstrated, however, a single administered antibiotic drug is not effective, but rather a mixture is [63]. This study also reported that only minor amounts of the administered drugs could be found in brain parenchyma. In particular, only 3% of metronidazole was found by LC-MS, while all other antibiotics were below detection range. This indicates that the drugs cannot exert a direct effect on pathomechanisms localized in the brain. Interestingly, Zarrinpar and colleagues described that oral gavage of C57BL/6 mice with antibiotics (ampicillin 100 mg/kg, vancomycin 50 mg/kg, metronidazole 100 mg/kg, neomycin 100 mg/kg, and amphotericin B 1 mg/kg, every 12 h for maximally 30 days, [64]) not only altered the microbiome indicated by a decrease in Firmicutes and Bacteroidetes. It consequently also affected bacterial metabolites, namely decreasing luminal short-chain fatty acids (SCFA) such as butyrate, and the secondary bile acid pool. This enforced the colonocytes of the hosts gut to shift from metabolizing SCFA towards glucose consumption and by this altered glucose homeostasis. For example, blood glucose levels were reduced both after short and extended fasting in the mice. The same observation was made in our study, where a reduction of blood glucose level of about 25% was observed in animals that were sacrificed 3 h after entering the period of rest where no food intake was expected. The elevated glucagon and the decreased insulin level furthermore underlined the anti-diabetic effect, even if the latter did not get statistically significant. A strong correlation of reduction of SCFA-producing bacteria was found in adult wild type mice receiving fecal transplantation from old donor mice in a recent publication [65]. This went along with, for example, an ageing-like phenotype of microglia in the CNS of the animals and could potentially hint at a deleterious effect of the observed age-related change in the microbiome. However, our study is based on a disease model, which comprises a completely different baseline condition.

To understand what aspect of the anti-diabetic effect of the antibiotics cocktail might be involved in ameliorating behavioral impairment of the 5xFAD mice, we investigated two exemplary pathways that are closely linked to glucose metabolism/insulin signaling: the XBP-1-linked ER-stress response and expression of RAGE. XBP-1 splicing and thereby activation of the transcription factor probability was considered as it has previously been identified as an alpha-secretase gene expression enhancer [36]. Stimulation of alpha-secretase ADAM10 expression and by this elevation of enzymatic activity could have explained the reduced Aβ deposition and in consequence improved the cognitive function of the mice (e.g., as shown by the pharmacological inducer acitretin, [27]). However, neither the amount of XBP-1 derived from the unspliced mRNA nor the protein derived from the spliced mRNA was affected in the brains of antibiotics-treated 5xFAD mice. In contrast, the RAGE protein amount was decreased. This receptor is able to transport Aβ peptides over the blood–brain barrier from the periphery, and thereby leads to an increase in cerebral amyloid load. Its blockade therefore has been assumed to be of therapeutical value [66,67]. This result corresponds with a study using type two diabetic male db/db mice [68]: chronic administration of antidiabetic drugs such as metformin or glibenclamide decreased Aβ influx across the blood–brain barrier by decreasing RAGE abundance.

In sum, we here provide evidence that antibiotics might elicit a beneficial effect on AD pathology by their anti-diabetic potential and the subsequent drop in the influx of Aβ. This once more demonstrates the complexity of studies on the involvement of the microbiota in non-gut disorders. The presence and composition of the commensal community has a multitude of systemically relevant effects on the host that in second line may affect molecular mechanisms at the border to or within the brain.

## Data Availability

Data is contained within the article or Supplementary Material.

## Supplementary Materials

The following are available online at https://www.mdpi.com/article/10.3390/microorganisms9040815/s1, Figure S1: Organ weights and colon length of 5xFAD mice treated with antibiotics or probiotics.
