# Supplementary material for: Impact of Gut Microbiome Manipulation in 5xFAD Mice on Alzheimer’s Disease-Like Pathology
Source: Microorganisms. 2021 Apr 13;9(4):815. doi: 10.3390/microorganisms9040815 (PMC8069338; doi:10.3390/microorganisms9040815)
Supplement: Supplementary file 1 [file microorganisms-09-00815-s001.pdf]

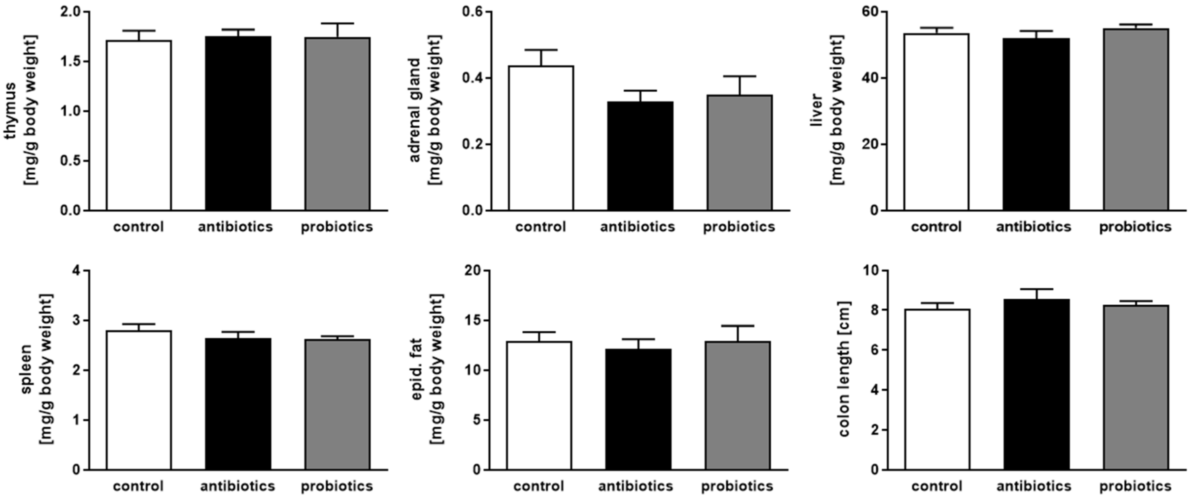

Suppl. Figure 1: Organ weights and colon length of 5xFAD mice treated with antibiotics or probiotics

Male mice (n=8 for control, n=7 for anti- or probiotics) were sacrificed after 14 weeks of treatment via drinking water at an age of 18 weeks. The respective organs were dissected, weighed and normalized for body weight. Colon was dissected and length from caecum ampulla to the rectum measured. Statistical analysis was performed by one-way ANOVA with Tukey's post-test. No significant differences occurred. For epididymal fat, two values had to be eliminated due to technical reason (one from control, one from probiotics group).
